# Optimizing the Calibration Error of Refraction Angles in Ultrasonic Angle Beam Testing

**DOI:** 10.3390/s20051427

**Published:** 2020-03-05

**Authors:** Zhihui Cai, Zhangmin Jin, Linyi Zhu, Yuebing Li, Yuebao Lei, Zengliang Gao

**Affiliations:** 1 Institute of Process Equipment & Control Engineering, Zhejiang University of Technology, Hangzhou 310032, China; zhihui.cai@zjut.edu.cn (Z.C.); zeroyi95@163.com (L.Z.); ylei@zjut.edu.cn (Y.L.); zlgao@zjut.edu.cn (Z.G.); 2 Wenzhou Special Equipment Inspection and Research Institute, Wenzhou 325800, China; 58658403@163.com; 3 EDF Energy Nuclear Generation Ltd., Barnett Way, Barnwood, Gloucester GL4 3RS, UK; 4 Engineering Research Center of Process Equipment and Re-manufacturing of Ministry of Education, Zhejiang University of Technology, Hangzhou 310032, China

**Keywords:** ultrasonic testing, instrument calibration, weighted measurement, optimal weights, data fusion, ultrasonic angle beam

## Abstract

Ultrasonic testing is a useful approach for quantifying the flaws in mechanical components. The height of the flaws in ultrasonic angle beam testing is closely related to the calibration value of the probe refraction angle. In order to reduce the calibration error, some ignored data during the traditional calibration process are reanalyzed and fused to determine the refraction angle. Both arithmetical measurement fusion method and weighted measurement fusion method are applied and compared. Monte Carlo simulation is used to estimate the probability distribution of the refraction angle and obtain the optimal refraction angle weights. Experiments were carried out to verify the results of Monte Carlo simulation. The applicability of data fusion on refraction angles is investigated. It was found in the study that the data fused with the refraction angle is helpful for measuring the height of flaws.

## 1. Introduction

In-service inspection is an approach to assure the reliability of a structure in the nuclear power, aerospace, railway transportation and petrochemical industries [1], where harmful flaws must be accurately measured. Ultrasonic testing (UT) is a popular technique for measuring flaws. Flaw size is needed in structural integrity assessment and structural health monitoring [2,3]. However, measurement of flaw size has been associated with errors. Flaw size with low standard deviation is helpful for structural integrity assessment and structural health monitoring since stress intensity factor depends on the flaw size. Therefore, it is necessary to improve the accuracy of the flaw size.

The UT with pulse waves is based on the fact that pulse-echoes propagate through the testing object and are reflected at the interface of flaws or an object boundary [4]. The most common pulse-echoes are longitudinal and shear waves. The ultrasonic probe transmits the pulse signals to the testing object and receives the returned pulse signals. Generally, ultrasonic angle beam (UTA) is used to measure flaw size in weld joints since the common orientation of flaws in weld joints is vertical. If the ultrasonic straight beam is used to measure vertical flaws, the pulse waves will make it difficult to find the reflected amplitude of the flaws.

The main uncertainties of flaw size measurement in UTA include: Material parameters, flaw characteristics, operation parameters and calibrations [5]. Pulse-waves may interact with materials in various ways, such as material properties, geometry and incidence angle. The amplitudes received are strongly affected by flaws characteristics (e.g., the size, skew tilt and type). There is a near-field region in the ultrasonic field. Ultrasound pressures fluctuate in the near-field region which make the UT more uncertain. The near field length *N* can be calculated by [6]

(1) $$N=\frac{D^{2}f}{4c},$$

where *f* is the transducer frequency, *D* is the crystal diameter and *c* is the sound velocity of the test material. An inspector should try to avoid inspecting flaws in the near-field in UT. An important factor about accurate measurement of a flaw size is calibration error. The calibration error will eventually affect the measurement error in the UT.

Probability distribution estimates of the size of flaws in ultrasound technology can be obtained through experiments and Monte Carlo simulations. Several studies were available for estimating the probability distributions of flaw size by model assisted methods and use these distributions to calculate the structural integrity of equipment [7,8]. If the flaw size is estimated accurately, the structural integrity of the equipment can be calculated accurately.

**Figure 1:**
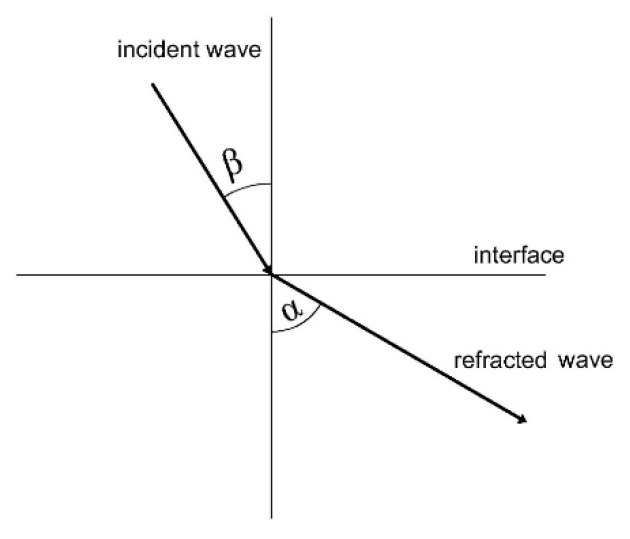
Snell’s law for refraction angle analysis.

There have been many studies conducted to improve the accuracy of flaw characteristic in UT. For example, F. Bettayeb suppressed noise to enhance flaw location by wavelet transform [9]; C. Zhou developed the probability-based diagnostic imaging approach to improve the accuracy [10]; L. Gandossi, J. He and J. Yang used Bayesian updating to improve accuracy [7,11,12]; J. Zhang used the scattering-matrix-based approach to increase sensitivity in ultrasonic arrays testing [13]; L. De Marchi used the warped frequency transform technology to overcome the difficulties associated with wave arrival time detection [14,15]. J. Rose introduced the refraction factor of ultrasonic wave in solid media [16]. Referred from Figure 1, the refraction angle can be calculated by

(2) $$\frac{c_{1}}{c_{2}}=\frac{\sin\alpha }{\sin\beta },$$

where *c*_1_ is the sound velocity of refracted wave, *c*_2_ is the sound velocity of incident wave, *α* is the refraction angle and *β* is the incident angle. The sound velocity is related to material characteristics and temperature [17]. Therefore, the refraction angle is affected by material properties, temperature and incident angle. It should be noted that an accurate refraction angle will be beneficial to characterize the defects. Since the manufacturing error of the incident angle is inevitable, the refraction angle should be calibrated for each probe and for each type of materials. Therefore, it is possible to improve the accuracy of defect detection by reducing the errors in the refraction angle calibration.

In this paper, the probability distribution of key parameters in the calibration is obtained by experiments. The optimal weights were calculated by Monte Carlo simulation. The probability distributions of the refraction angle were optimized by the optimal weights. Optimized refraction results are used to measure flaws. The applicability of data fusion was analyzed. The measurement accuracies were improved after data fusion. The improvement is obvious for big flaws or larger refraction angle probes. This paper is organized as follows. Firstly, the methods and experiments are described in Section 2. Secondly, the results are counted and discussed in Section 3. Finally, the conclusions are drawn in Section 4.

## 2. Methods

### 2.1. Methods of Calibration

There are different calibration blocks in many standards [18,19,20]. Calibration methods are different for different calibration blocks. In this study, we used a CSK-IA calibration block and a CSK-IIA-2 calibration block in a Chinese standard NB/T 47013-2015 [18]. These calibration blocks were made of normalizing 20# forged steel. The parameters which need to be calibrated are sound velocity, time base, probe index and refraction angle.

**Figure 2:**
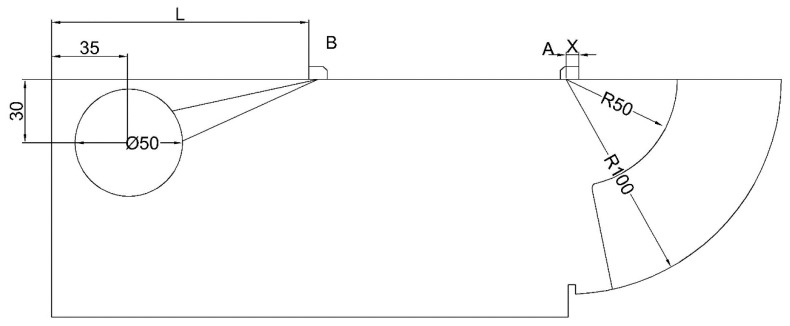
Calibration by CSK-IA block.

**Figure 3:**
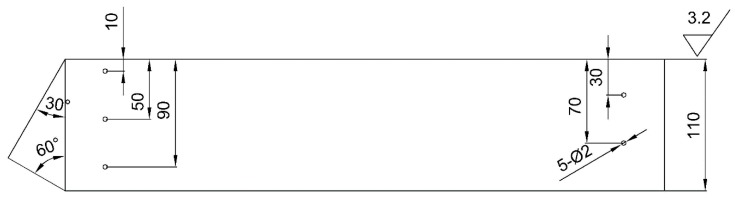
Calibration by CSK-IIA-2 block.

The pulse-echo-type of ultrasonic instruments only obtain the time difference between transmit pulse waves and receive pulse-echoes. Sonic path distance *S* can be calculated by

(3) $$S=\frac{c\times (t-\tau )}{2},$$

where *t* is the time difference between transmitting pulse wave and receiving pulse-echo, *c* is the sound velocity and *τ* is the delay time. There are many reasons for the delay time. Such as the pulse-echo propagation in probe, acoustoelectric conversion and so on. Usually, inspectors use a parameter that named time base to describe the delay time. When an inspector uses the CSK-IA calibration block in calibration, there are two steps. For step one, the inspector places the probe near position A, as shown in Figure 2, and finds the position which has maximum response for the arc; then the parameters are

(4) $$\tau =2\times t_{50}-t_{100},$$

(5) $$c=\frac{50}{2(t_{100}-t_{50})},$$

where *t*_50_ is the propagation time of the pulse wave between the probe and arc with a radius of 50 mm and *t*_100_ is the propagation time of the pulse wave between the probe to the arc with a radius of 100 mm. The distance between the front side of the probe and the end of arc with radius of 100 mm is measured by ruler, and the probe index can be calculated. For step two, the inspector places the probe near position B, as show in Figure 3, and finds the position which has the maximum reflex amplitude for the circular hole. Inspectors usually use the K as the tangent value of the refraction angle to represent the refraction angle in engineering. Once the inspector finds the maximum response of the circular hole, there are two ways to obtain the refraction angle, i.e., by ruler or by instrument. For the ruler, the value of K can be calculated by

(6) K=[(L−35)+X]/30,

where *L* is the distance from the front side of the probe to the end of the CSK-IA (DFSP) block and *X* is the probe index calibrated in step one. For the instrument, the value of *K* can be calculated by

(7) $$K=\left\{\sqrt{{\left[c\times (T_{H}-\tau )/2+25\right]}^{2}-900}\right\}/30,$$

where *T*_H_ is the time of the pulse wave in propagation between the probe and the hole in the CSK-IA block.

The distance amplitude correction (DAC) technique is the most commonly used technique for ultrasonic waves attenuation in UT. It firstly scans artificial reflectors with different sound path distances in the CSK-IIA-2 calibration block. Then, it records their maximum reflection amplitude. Finally, the data of different sound path distances and amplitudes are fitted into a curve displayed on the screen of the instrument.

### 2.2. Methods for Obtaining Flaw Height

The common techniques for the measurement of flaw size are the maximum echo height techniques and probe movement sizing techniques [21]. The maximum echo height technique typically measures flaws (e.g., volume defects) that have a small effect on shape and orientation with respect to the received amplitude. Probe movement sizing technology typically measures flaws (e.g., planar defects) where the shape and orientation have a greater effect on the received amplitude. In addition to these two techniques, there are special quantization techniques that can be used to obtain accurate dimensions, e.g., tip diffraction techniques and the mode conversion techniques.

In this study, we focused on planar flaws which are more severe in structural integrity. The best accuracy in height of flaws is achieved by obtaining and analyzing signals for crack tip [19]. The flaws are classified as top-surface connected flaws (TSCF), bottom-surface connected flaws (BSCF) and embedded flaws (EF) [20].

**Figure 4:**
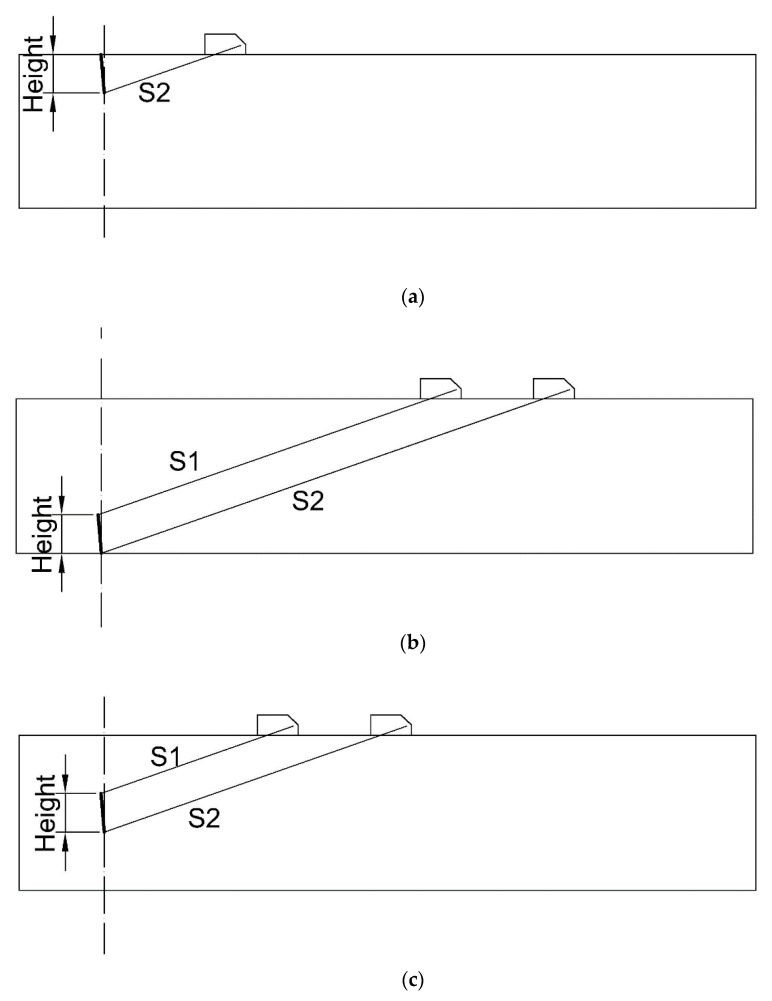
Measure flaw height of (**a**) top-surface connected flaws (TSCF), (**b**) bottom-surface connected flaws (BSCF) and (**c**) embedded flaws (EF).

For TSCF, the geometrical relationship of the measurement endpoint is shown in Figure 4a when the single traverse technique is used. The height of the flaw can be calculated as

(8) $$\begin{array}{cc} h & =S_{2}\times \cos\alpha \\ & =c\times (t_{2}-\tau )/2\times \cos\alpha , \end{array}$$

where *t*_2_ is the propagation time of the pulse wave between the probe and the lower endpoint of the flaw and *α* is the refraction angle of ultrasonic in the testing object. The measured value is related to the refraction angle, time base and sound velocity which are obtained by calibration.

For BSCF and EF, using the single-conductor technology, the geometric relationship between the measurement endpoints is shown in Figure 4b,c. The height of flaw can be calculated as:

(9) $$\begin{array}{cc} h & =(S_{2}-S_{1})\times \cos\alpha \\ & =c\times (t_{2}-t_{1})/2\times \cos\alpha , \end{array}$$

where *t*_1_ is the propagation time of the pulse wave between the probe and the upper endpoint. The measured value is related to the refraction angle and sound velocity which obtained by calibration.

From Equations (8) and (9), it is known that the accuracy of the refraction angle is a key factor in the accuracy of the flaw height. If the refraction angle error during calibration is large, the measurement results after calibration may have more errors. It can be known from Equation (9) that when measuring BSCF or EF, the time base error has nothing to do with the measurement result.

### 2.3. Optimizing the Values of the Refraction Angle in the DAC Calibration Procedure

Some information in the DAC calibration procedure helps to optimize the refraction angle. The inspector looks for the probe position that receives the maximum response from the artificial reflector. Normally, it only records amplitude and sonic path distance during calibration, ignoring the depth of the artificial reflector, the relationship between the acoustic wave path of the artificial reflector and the refraction angle of the probe. The refraction angles can be calculated from the depths and the sonic paths of artificial reflectors, then refraction angles that are calculated can be fused using mathematical methods.

The errors can be described with variance of measured values. Data fusion technology can improve the accuracy of calibration values. There are many methods for data fusion, for example, arithmetical measurement fusion (AMF), weighted measurement fusion (WMF) and Kalman filtering. Kalman filtering usually deals with dynamic data. AMF is the easiest method for data fusion, while its variance, typically, is not the smallest. WMF is the most efficient method in data fusion. The variance of probability distribution is changed with weight changed by WMF. There is an optimal weight that obtains minimal variance.

Define the measured values as *D_i_* (*i* = 1, 2, …, *n*) for one object:

(10) $$\bar{D}=\sum_{i=1}^{n}W_{i}D_{i},$$

where $\bar{D}$ is the weighted mean and $\sum_{i=1}^{n}W_{i}=1$. The variance

(11) $$\begin{array}{l} \sigma^{2}=E\left\{{\left[D-E(D)\right]}^{2}\right\}\\ =E\left\{[\sum_{i=1}^{n}(W_{i}D_{i}-W_{i}\bar{D}){]}^{2}\right\}\\ =E\left\{[\sum_{i=1}^{n}{\left(W_{i}(D_{i}-\bar{D})\right]}^{2}\right\}\\ =\sum_{i=1}^{n}\sum_{j=1}^{n}W_{i}W_{j}COV(D_{i},D_{j}), \end{array}$$

where *COV*(*D_i_*, *D_j_*) are the covariances. The Lagrange multiplier method can be used to find the optimal weights. The Lagrange function is

(12) $$La=f(W_{i})+\lambda \varphi (Wi),$$

where $f(W_{i})=\sum_{i=1}^{n}\sum_{j=1}^{n}W_{i}W_{j}\mathrm{cov}(D_{i},D_{j})$ and $\varphi (W_{i})=\sum_{i=1}^{n}W_{i}-1$. When $\partial La/\partial W_{i}$ and ∂La/∂λ are existence and continuity, the minimizes variance is existence and the optimal weights can be calculated by

(13) $$\left\{\begin{array}{l} \partial La/\partial W_{i}=0\\ \partial La/\partial \lambda =0 \end{array}\right\}.$$

### 2.4. Experiments

**Figure 5:**
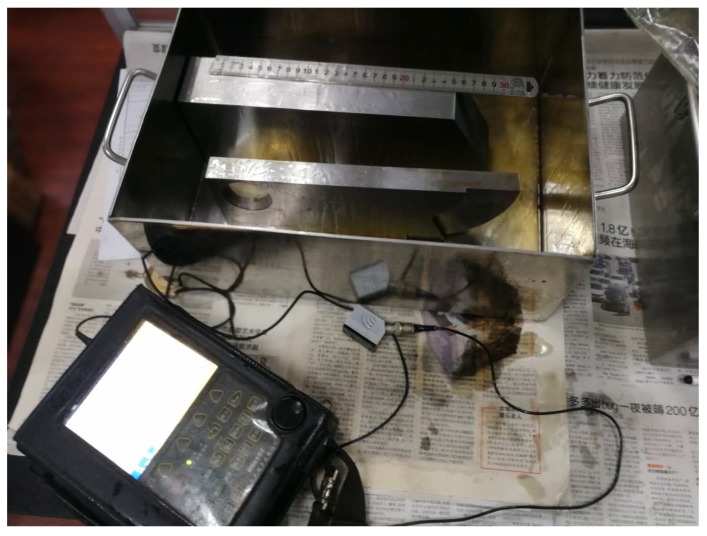
The experimental environment.

In order to find the probability distribution of key values in the calibration, three experiments were performed in this study. In these experiments, the ultrasonic instrument was HS610e, the nominal frequency of the probes was 2.5 MHz, the crystal size of the probes were 10 by 16 mm, the nominal refraction angles of probes were 1.0, 1.5, 2.0 and 2.5, respectively. The base setting of the ultrasonic instrument was a sound velocity of 3240 m/s and a time base of 10 µs. The experimental environment is shown in Figure 5. The three calibration experiments were as follows.

Experiment 1 (EXP1): The probes, placed near position A in Figure 2, found the position which had the maximum responses for the arc with radii of 50 mm and 100 mm. Next, the sonic path distance and probe index were recorded. These procedures were repeated for several times. According to Equation (3), the propagation time of the pulse waves can be calculated as:

(14) t=2×S/3.24+10

Experiment 2 (EXP2): The probes, placed near position B in Figure 3, found the position which had the maximum reflex amplitude for the circular hole. Then the sonic path distance and DFSP were recorded at the same position. These procedures of finding and recording were repeated for several times. In the end, the sound path distances were converted into propagation time by Equation (14).

Experiment 3 (EXP3): Firstly, the side-drilled holes (SDH) in CSK-IIA-2 block were scanned. Secondly, the sonic path distances and depths of SDH were recorded when probe obtain the maximum reflex amplitudes. These procedures of scanning and recording were repeated for several times. In the end, the sound path distances were converted into propagation time by Equation (14).

**Figure 6:**
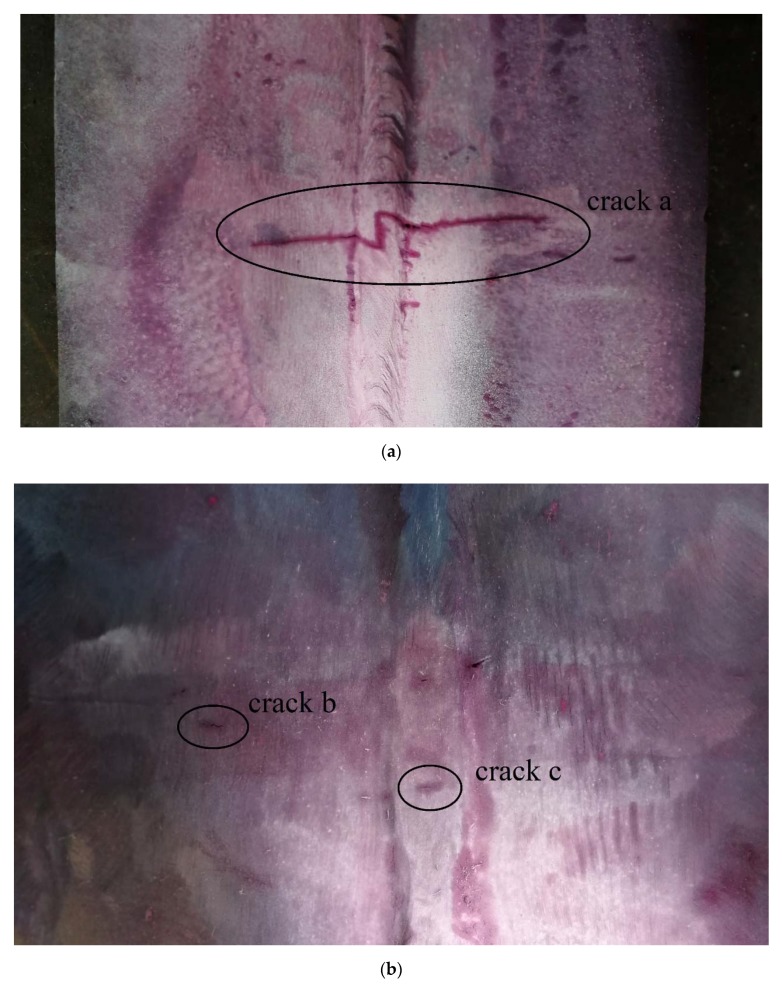
The test block with (**a**) crack *a* and (**b**) crack *b* and crack *c*.

Experiment 4 (EXP4): In order to find the probability distribution of the key parameters in the measurement, two surface crack test blocks were used in the experiment. The test blocks with three cracks are shown in Figure 6. To better show flaws, penetrant testing was performed for all the cracks. Tip diffraction techniques are preferred for measuring the height of crack-like flaws [18,22]. Therefore, they were used to measure the cracks from top surface and bottom surface with different probes. Although some literature has shown that the K1 refraction angle probe has higher accuracy and some standards prefer to use it [18,23], it is difficult to measure flaws near the probe due to the near field. Therefore, we used probes with different refraction angles. The base setting of ultrasonic instrument was the same as those of EXP1–3.

Experiment 5 (EXP5): The purpose of this experiment is to compare the results with Monte Carlo simulation and actual measurement. Firstly, some data were supplemented as EXP1–3 and combined with the results of EXP4. Secondly, *K* in the calibration were fused by weights which were obtained in Monte Carlo simulation and calibration data which were supplemented in the DAC calibrations. Thirdly, the flaw sizes were calculated. Fourthly, the cracks were grinded until the cracks disappeared. Finally, the depth of the last grinding when cracks were displayed (HMIN) and the grinding depth when the cracks disappear (HMAX) were recorded. Therefore, the actual crack heights were between HMIN and HMAX.

### 2.5. Monte Carlo Simulation

**Figure 7:**
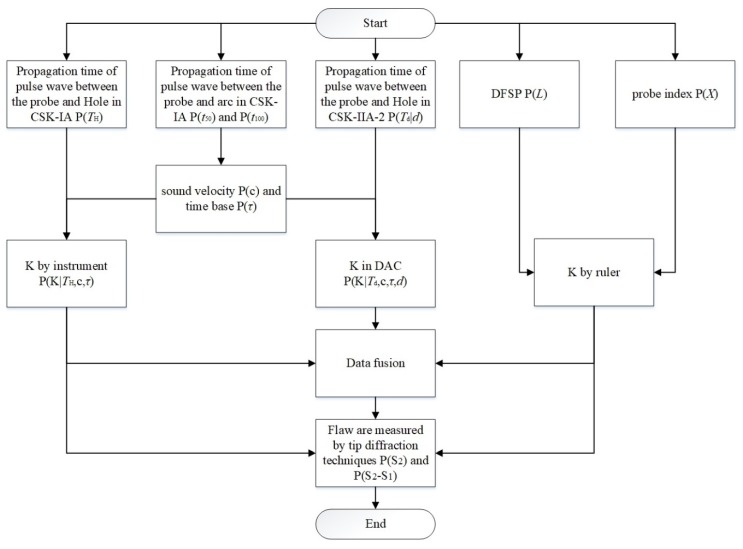
Procedure of Monte Carlo simulation.

The probability distributions of the key parameters were obtained. Then we used Monte Carlo simulation to calculate the distribution of calibration parameters. The procedure of the Monte Carlo simulation is shown in Figure 7. Firstly, the random numbers of *X*, *t*_100_ and *t*_50_ were generated. Secondly, time base *τ* and sound velocity *c* were calculated by Equations (4) and (5). Thirdly, the random numbers of *T*_H_ and *L* were generated. Fourthly, the K by ruler were calculated by Equation (6) and K by instrument were calculated by Equation (7), the K with difference depth of holes in DAC calibrations were calculated by:

(15) $$K=\left\{\sqrt{{\left[c\times (T_{d}-\tau )+r\right]}^{2}-d^{2}}\right\}/d,$$

where *r* is the radius of SDHs, *d* is the depth of SDH and *T_d_* is the propagation time of the pulse wave between the probe and SDH. Fifthly, random numbers of S_2_ and (S_2_–S_1_) were generated. In the end, the height of the flaw can be calculated by Equations (8) and (9).

## 3. Results and Discussion

### 3.1. Results of the Experiment

The software Matlab2018a was used in the analyses. KS-test was used to test the obtained data in EXP1–2. The null hypothesis is that the data follow the normal distribution. A P value greater than 0.05 indicates acceptance of the null hypothesis. Then the probability distribution of the data could be approximated as a normal distribution. The statistical results and P values in the KS-tests are shown in Table 1. From Table 1, it can be seen that all P values are greater than 0.05. Therefore, the distributions of the data could be approximated as normal distributions. The standard deviation of the measured values increases as the angle of refraction increases, except for *X*. The Pearson correlation coefficients are shown in Table 2. *t*_100_ and *t*_50_ and *L* and *T*_D_ have significant correlations. The means and standard deviations of sound velocity, time base, K by ruler and K by instrument are shown in Table 3. From Table 3, it can be seen that the mean values of the sound velocity for the different probe in calibration are different. The sound velocity is determined by the material itself, which is not affected by the refraction angle of the probe. The calibration difference of the different probes may be caused by a calibration error and an arc error in CSK-IA block. Therefore, it is necessary to average the sound velocities measured by all the probes of a material to obtain accurate results.

The times of measurement is 30 in EXP3. The results which have been converted from sonic path distance to propagation time in EXP3 are shown in Table 4. All P values in KS-test are greater than 0.05. It can be found from the results that the measurement results with smaller refraction angles are more accurate.

Table 5 shows the results of conversion from sonic path distance to propagation time in EXP4. All the P values of the propagation time are greater than 0.05. The grinding depth in the experiments is shown in Table 6. In the experiment, crack c was difficult to find since the size of the flaw was small and the flaw was in the near field. For the same reason, the tip diffraction waves of crack b were difficult to determine. Therefore, only the K2.5 probe could faintly determine the diffraction waves from bottom-surface. From the results, it can be concluded that a small refraction angle has no advantage since the tip diffraction wave is hard to find in the near field. It is easy to misjudge the position of the tip diffraction wave in the near field.

### 3.2. Results of Monte Carlo Simulation of Data Fusion K

According to the results of the experiments, the random numbers of *X*, *t*_100_, *t*_50,_ *T*_H_, *L*, *T_d_*, S_2_ and (S_2_-S_1_) were generated as normal distributions. The means and standard deviations were the same as the results of experiments which are shown in Table 1 and Table 4. The Pearson correlation coefficients of random numbers of *t*_100_ and *t*_50_ and that of *T*_H_ and *L* are the same as those in Table 2. Considering the errors in experiments, the expectation values and means are usually not equal. Therefore, it was assumed that the expectation value of *T*_d_ can be calculated by

(16) $$E(T_{d})=\frac{d\times \sqrt{MK^{2}+1}-r}{c}+\tau ,$$

where *MK* is the mean of *K* by instrument and *K* by ruler in the experiments. The instrument specification states that the shear wave propagates at a speed of 3240 m/s in low-carbon steel. Therefore, in the simulation, *c* was set to be equal to 3240 m/s.

The optimal weights in data fusion K are shown in Table 7. Calibration K by ruler is independent of calibration K by instrument and in DAC. Therefore, calibration K by ruler has a high weight. From Table 7, it can be found that the deeper the SDH, the higher the weight of the data.

**Figure 8:**
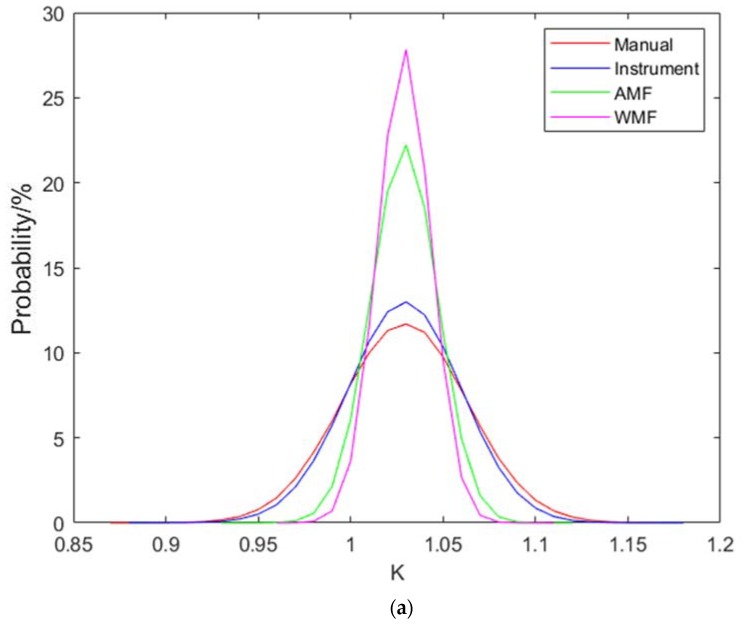

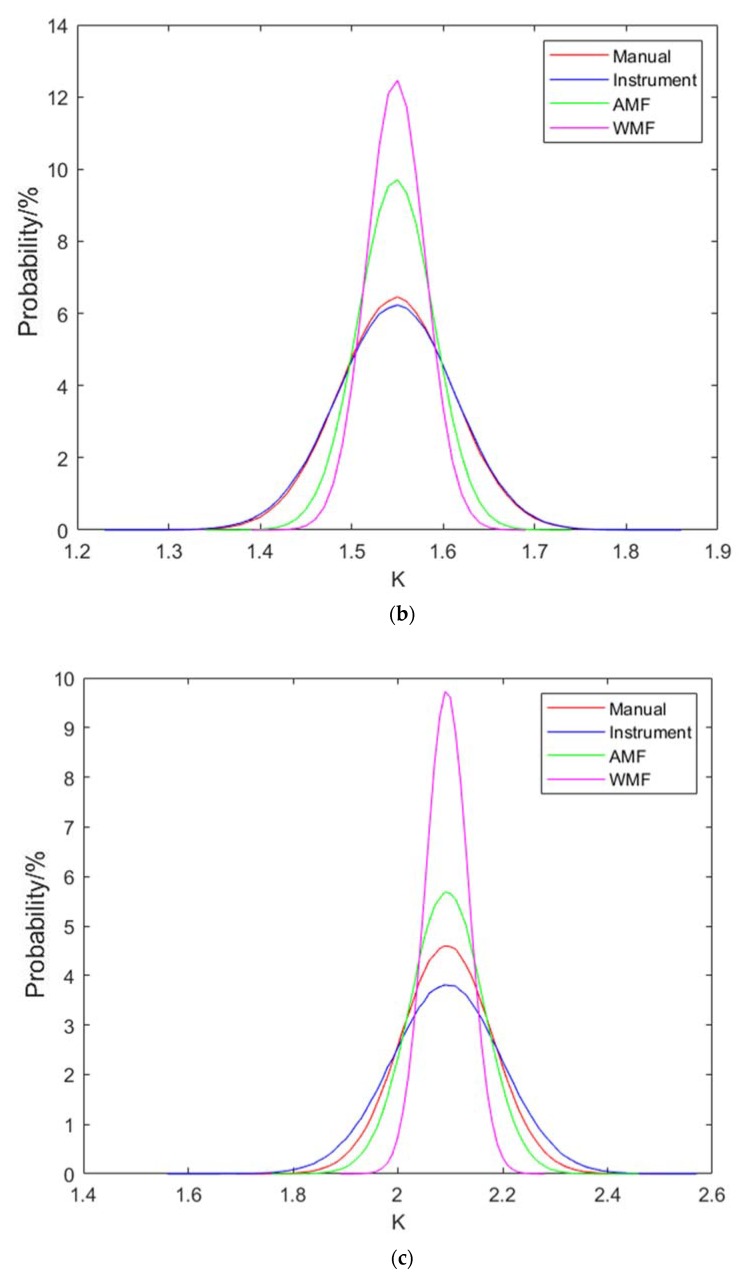

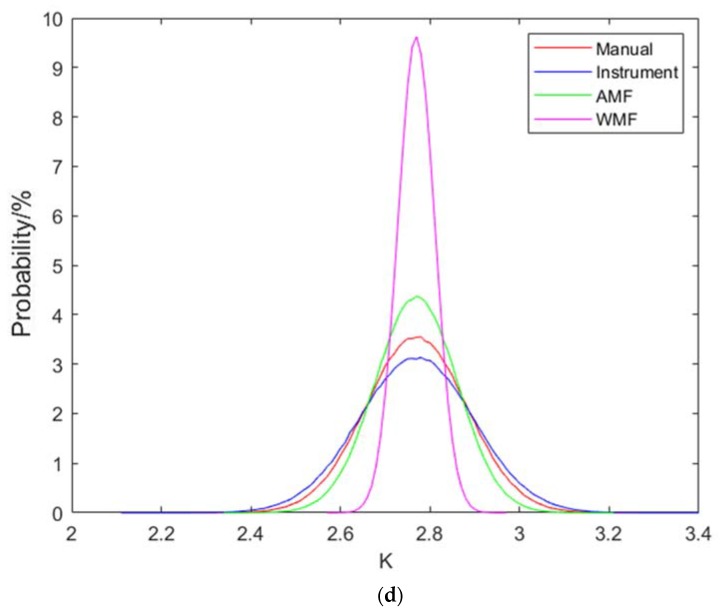
Results of fusion K of (**a**) K1, (**b**) K1.5, (**c**) K2 and (**d**) K2.5 probes.

The results of data fusion K are shown in Figure 8. These figures show that data fusion is effective for improving the refraction angle accuracy. WMF is better than AMF. Probes with large refraction angles are optimized better than those with smaller refraction angles because probes with large refraction angles have larger errors in calibration.

**Figure 9:**
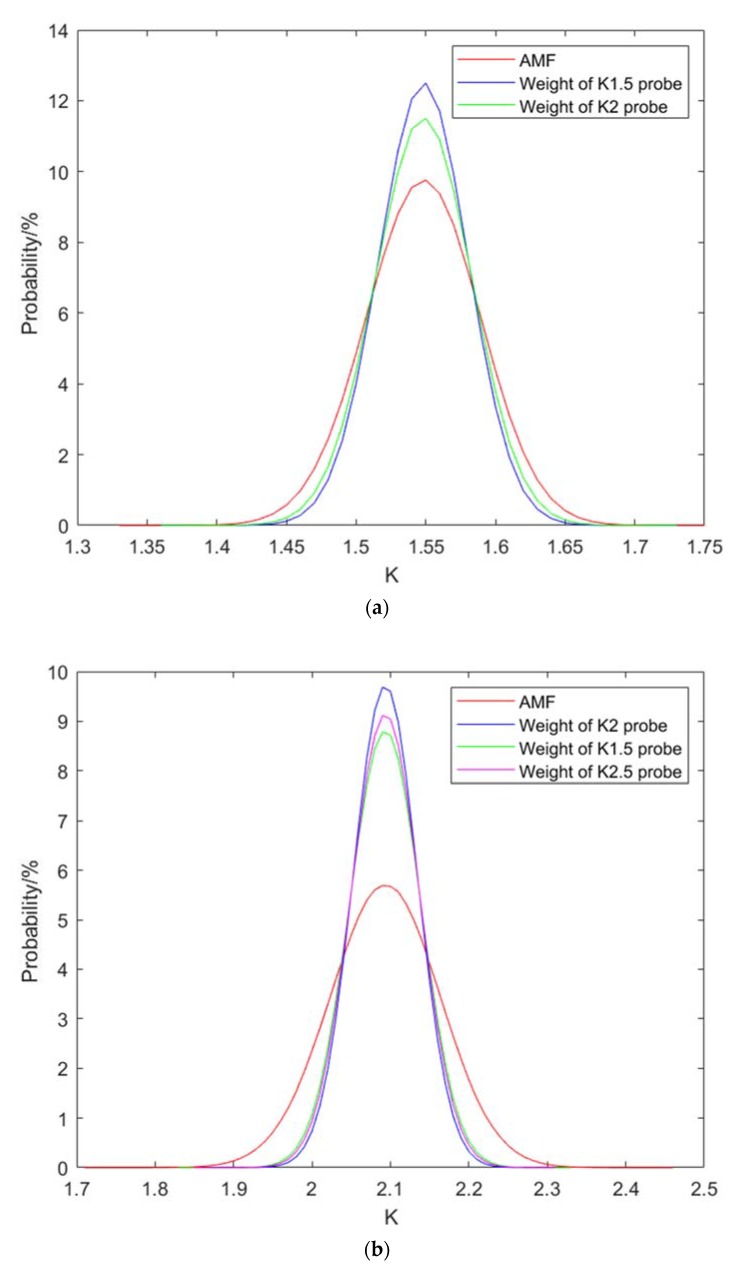

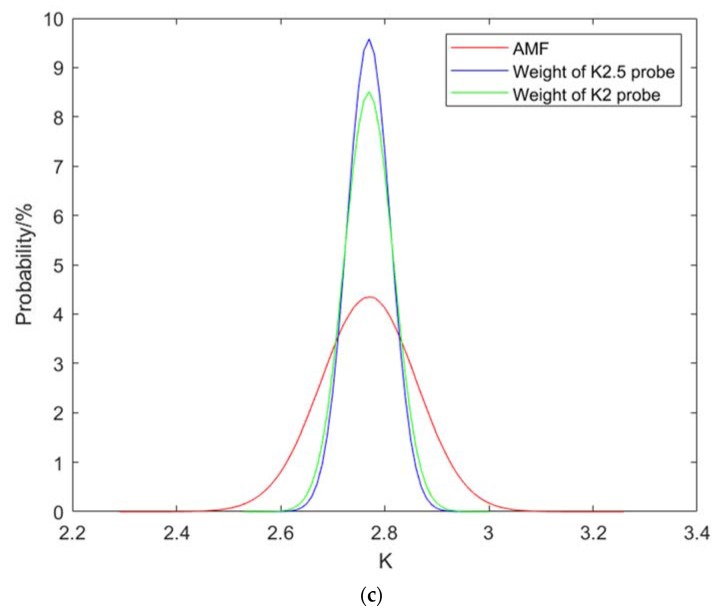
Results of fusion K of (**a**) K1.5, (**b**) K2 and (**c**) K2.5 probes by another probe weights.

From Table 7, it can be seen that the probes with different refraction angles have different optimal weights. In fact, the true refraction angle of probe is unknown. Hence, the optimal weight is hard to be determined. Therefore, we tried to fuse a certain probe with the optimal weight of different probes. The probe with a refraction angle of K1.5 was fused by the optimal weight of the K1 probe and the optimal weight of the K2 probe. The probe with a refraction angle of K2 was fused by the optimal weight of the K1.5 probe and the optimal weight of the K2.5 probe. The probe with a refraction angle of K2.5 was fused by the optimal weight of the K2 probe. The fusion result is shown in Figure 9. It can be seen from the results that the optimal weight fusion of other probes can also achieve good results. Therefore, the difference in the actual refraction angle between the probes with the same nominal refraction angle is small. As a result, the difference in refraction angle between the probe and the probe with the same nominal refraction angle is negligible.

### 3.3. Results of Monte Carlo Simulation and Experiments for Measurement Flaw Size

From Equations (8) and (9), the height of the flaw can be expressed as:

(17) h=RS×RK,

where *RS* is a random number about the sonic path distance and *RK* is a random number about the refraction angle. Combining Equations (7), (8) and (16), the *RS* and *RK* are dependent on *τ*, therefore, *RS* and *RK* are dependent on each other. However, *RS* is independent of *τ* in Equation (9), therefore, *RS* and *RK* are independent of each other when BSCF or EF are measured. When *RS* and *RK* are independent random variables, then:

(18) $$\begin{array}{cc} E(h) & =E(RS\times RK)\\ & =COV(RS,RK)+E(RS)\times E(RK)\\ & =E(RS)\times E(RK), \end{array}$$

(19) $$\begin{array}{l} D(RS\times RK)=E(RS^{2}\times RK^{2})-{\left[E(RS\times RK)\right]}^{2}\\ =E(RS^{2})\times E(RK^{2})-{\left[E(RS)\times E(RK)\right]}^{2}\\ =\left[D(RS)+E{\left(RS\right)}^{2}\right]\times \left[D(RK)+E{\left(RK\right)}^{2}\right]-{\left[E(RS)\times E(RK)\right]}^{2}. \end{array}$$

Therefore, as the standard deviation of *RK* decreases by data fusion, the standard deviation of heights decreases. The degree of decrease is affected by $\left[D(RS)+E{\left(RS\right)}^{2}\right]$.

**Figure 10:**
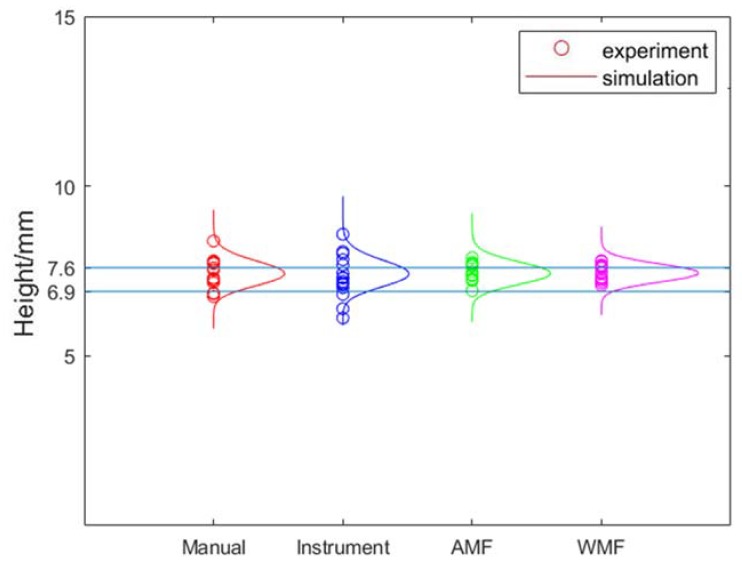
The effect of measuring crack *a* from bottom-surface by K2.5 Probe.

**Figure 11:**
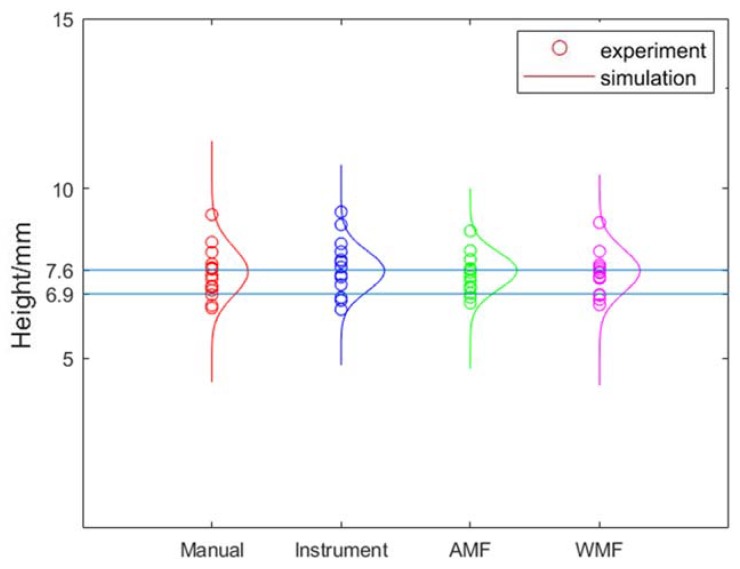
The effect of measuring crack *a* from top-surface by K2.5 Probe.

The means of the flaw height of Monte Carlo simulation and experiments are shown in Table 8. The standard deviations of the results of the Monte Carlo simulation and experiments for the measurement of flaws size are shown in Table 9. From Table 8 and Table 9, it can be seen that the results of Monte Carlo simulations are similar to those of experiments. From Table 8, it can be seen that most of the means are between HMIN and HMAX. Therefore, the results of experiments and Monte Carlo simulations are in good agreement with the actual height of flaws. The results of Monte Carlo simulation were analyzed since the number of samples in experiments were small and the results of Monte Carlo simulation and experiments were similar. When BSCF are measured, *RS* and *RK* are independent of each other. When the refraction angle is small, the K value calibrated by ruler or by instrument is accurate. Therefore, the effect of data fusion optimization is not obvious. The similar results can be found in Table 9. The standard deviations of WMF are small than AMF when BSCF are measured. The bigger the refraction angle, the greater difference between WMF and AMF. When K2.5 probe was used, the effect was obvious, as shown in Figure 10. When BSCF are measured, the *RS* and *RK* depend on each other. The optimal weight of K is calculated with the covariance matrix in Equations (10)–(13). Therefore, when *RS* and *RK* depend on each other, the optimal weights were changed by multiplication, therefore, WMF is not applicable. It can be found from Table 9 that AMF is better than WMF. When K2.5 probe was used, the effect was obvious, as shown in Figure 11. Compared with the measurement of crack a using a K2.5 probe and the measurement of crack b using a K2.5 probe, the E (*RS*) in crack *a* is larger than the E (*RS*) in crack *b*. The optimization effect of WMF on crack a is better than that on crack b. Therefore, it can be concluded that WMF is more effective for larger flaws.

## 4. Conclusions

This paper proposes two new approaches for reducing errors in instrument calibration, namely AMF and WMF. The optimal weights were obtained in this paper. The data of deeper SDH have a higher weight. The AMF applies to all flaws. However, WMF only applies to defects where the measured path distance is independent of time bases such as BSCF and EF. The optimal weights are calculated by Monte Carlo simulation.

The K1 probe is usually used to receive the primary wave to measure the defect height. However, due to the near field, the K1 probe has no advantage in measuring the defect height near the probe. In general, inspectors use larger refraction angle probes or receive multiple reflected ultrasound waves to measure the height of flaws near the probe. When using ultrasonic waves with multiple reflections, the condition of the reflecting surface will affect the measurement accuracy. When using a larger refraction angle probe, its K calibration error is larger. Data fusion can reduce the calibration error of K. AMF can be used to measure TSCF, WMF can be used to measure BSCF and EF. After data fusion, the measurement accuracy is improved. In the measurement of severe flaw structures, the improvement effect is very good.

Digital ultrasound instruments are commonly used in engineering. If these methods are embedded in the instrument program, measurement accuracy can be improved without reducing efficiency. This study only proposes two fusion K methods. The optimal weights are different for different refraction angles. The refraction angle is slightly different for the same nominal value. Therefore, the optimal weight calculated in this paper can be directly used without considering the difference of each probe with the same nominal refraction angle.

## Figures and Tables

**Table 1:** Results of Experiment 1 (EXP1) and Experiment 2 (EXP2).

Item	Probe	Mean	Standard Deviation	Times	*P* Value for KS-Test
*t*_100_	K1	68.22 µs	0.31	39	0.73
	K1.5	68.67 µs	0.35	17	0.8
	K2	70.03 µs	0.84	23	0.98
	K2.5	68.85 µs	1.15	41	0.89
*t*_50_	K1	37.3 µs	0.3	39	0.7
	K1.5	37.84 µs	0.4	17	0.83
	K2	39.21 µs	0.9	23	0.9
	K2.5	38 µs	1.1	41	0.79
*X*	K1	9.51 mm	0.71	39	0.89
	K1.5	8.49 mm	0.62	17	0.97
	K2	9.39 mm	0.74	23	0.99
	K2.5	12.94 mm	0.91	41	0.65
*L*	K1	56.62 mm	0.72	39	0.49
	K1.5	73.22 mm	1.76	17	0.55
	K2	88.52 mm	2.48	23	0.93
	K2.5	106.06 mm	3.24	41	0.91
*T*_D_	K1	17.44 µs	0.27	39	0.64
	K1.5	25.56 µs	0.88	17	0.88
	K2	35.83 µs	1.44	23	0.98
	K2.5	45.7 µs	1.94	41	0.73

**Table 2:** Pearson correlation coefficients.

Probe	K1	K1.5	K2	K2.5
*t*_100_ and *t*_50_	0.96	0.98	0.99	0.99
*L* and *T*_D_	0.71	0.78	0.9	0.93

**Table 3:** Results of calibrations (mean/standard deviation).

Probe	c	τ	K by Ruler	K by Instrument
K1	3234.88/9.16	6.39/0.33	1.04/0.035	1.02/0.029
K1.5	3243.46/9.22	7/0.46	1.56/0.056	1.54/0.056
K2	3245.13/12.55	8.4/0.99	2.1/0.079	2.09/0.088
K2.5	3242.79/18.26	7.17/1.08	2.8/0.11	2.74/0.128

**Table 4:** Propagation time in Experiment 3 (EXP3) (mean/standard deviation), µs.

Depth	K1	K1.5	K2	K2.5
10	-	18.18/0.38	22.52/0.47	27.72/1.78
30	32.33/0.52	40.39/1.03	50.14/1.2	60.25/2.03
50	49.82/0.61	62.73/1.31	77.94/2.11	98.55/1.87
70	67.74/0.68	86.36/1.95	106.81/2.25	137.72/2.01
90	85.3/0.71	-	-	-

**Table 5:** Propagation time in EXP3 (mean/standard deviation), µs.

	*t*_2_	*t*_2_–*t*_1_	Times
K1 with crack a	-	6.33/0.82	15
K1.5 with crack a	16.07/0.73	8.21/0.77	15
K2 with crack a	19.47/0.81	9.8/0.77	15
K2.5 with crack a	20.93/0.52	13.53/0.44	15
K2.5 with crack b	-	2.84/0.31	6

**Table 6:** The grinding depths of crack.

	Crack a	Crack b	Crack c
HMIN	6.9	2.1	-
HMAX	7.6	2.7	1.2

**Table 7:** The optimal weights in data fusion K.

	Ruler	Instrument	10	30	50	70	90
K1	0.2179	−0.0943	0.0309	0.1516	0.2602	0.4337	-
K1.5	0.2828	−0.0256	-	−0.0108	0.1485	0.3121	0.2390
K2	0.4176	−0.2245	-	−0.0357	0.2021	0.2243	0.4163
K2.5	0.3549	−0.2454	-	−0.0074	0.0709	0.2997	0.5272

**Table 8:** The means of the flaws height of Monte Carlo simulation and experiments, (simulations/experiments), mm.

Crack	Probe	Location	Manual	Instrument	AMF	WMF
a	K1	Bottom-surface	7.1417/7.1846	7.1423/7.1467	7.1416/7.1467	7.1412/7.1448
a	K1.5	Bottom-surface	7.2201/7.18	7.2217/7.2192	7.2191/7.2106	7.2175/7.195
a	K1.5	Top-surface	7.09/7.0708	7.086/7.1012	7.0834/7.0442	7.0852/7.0299
a	K2	Bottom-surface	6.8489/6.835	6.8535/6.9414	6.848/6.8694	6.8437/6.8341
a	K2	Top-surface	7.7363/7.8083	7.7255/7.8431	7.7192/7.8047	7.7274/7.8675
a	K2.5	Bottom-surface	7.4517/7.4138	7.4551/7.3315	7.4503/7.4463	7.444/7.4507
a	K2.5	Top-surface	7.5789/7.4934	7.5706/7.5751	7.5655/7.4878	7.5686/7.4519
b	K2.5	Bottom-surface	2.5342/2.5105	2.5353/2.4641	2.5337/2.4832	2.5315/2.5369

**Table 9:** The standard deviation of the results of the Monte Carlo simulations and experiments, (simulations/experiments), mm.

Crack	Probe	Location	Manual	Instrument	AMF	WMF
a	K1	Bottom-surface	0.9377/0.9861	0.9362/0.9613	0.9318/0.9285	0.931/0.9264
a	K1.5	Bottom-surface	0.71/0.6907	0.712/0.7324	0.693/0.6947	0.6877/0.69
a	K1.5	Top-surface	0.7838/0.8326	0.7355/0.8558	0.7154/0.7189	0.7492/0.6897
a	K2	Bottom-surface	0.5825/0.5992	0.6043/0.6756	0.5665/0.5961	0.5454/0.5859
a	K2	Top-surface	0.9277/0.96	0.8052/0.9262	0.7686/0.836	0.8721/1.0377
a	K2.5	Bottom-surface	0.3617/0.4438	0.3897/0.6439	0.3265/0.2693	0.2612/0.2305
a	K2.5	Top-surface	0.7153/0.7198	0.5949/0.8554	0.5535/0.5595	0.6396/0.602
b	K2.5	Bottom-surface	0.2954/0.2828	0.2996/0.2972	0.2905/0.3082	0.2805/0.3136
